# Promising bioactive properties of quercetin for potential food applications and health benefits: A review

**DOI:** 10.3389/fnut.2022.999752

**Published:** 2022-11-30

**Authors:** Irtiqa Shabir, Vinay Kumar Pandey, Rafeeya Shams, Aamir Hussain Dar, Kshirod Kumar Dash, Shafat Ahmad Khan, Iqra Bashir, G. Jeevarathinam, Alexandru Vasile Rusu, Tuba Esatbeyoglu, R. Pandiselvam

**Affiliations:** ^1^Department of Food Technology, Islamic University of Science and Technology Kashmir, Pulwama, India; ^2^Department of Bioengineering, Integral University, Lucknow, Uttar Pradesh, India; ^3^Department of Biotechnology, Axis Institute of Higher Education, Kanpur, Uttar Pradesh, India; ^4^Department of Food Technology and Nutrition, Lovely Professional University, Phagwara, Punjab, India; ^5^Department of Food Processing Technology, Ghani Khan Choudhury Institute of Engineering and Technology (GKCIET), Malda, West Bengal, India; ^6^Division of Food Science and Technology, Sher-e-Kashmir University of Agricultural Sciences and Technology, Srinagar, Kashmir, India; ^7^Department of Food Technology, Hindusthan College of Engineering and Technology, Coimbatore, Tamil Nadu, India; ^8^Life Science Institute, University of Agricultural Sciences and Veterinary Medicine Cluj-Napoca, Cluj-Napoca, Romania; ^9^Animal Science and Biotechnology Faculty, University of Agricultural Sciences and Veterinary Medicine Cluj-Napoca, Cluj-Napoca, Romania; ^10^Department of Food Development and Food Quality, Institute of Food Science and Human Nutrition, Gottfried Wilhelm Leibniz University Hannover, Hannover, Germany; ^11^Physiology, Biochemistry and Post-Harvest Technology Division, ICAR-Central Plantation Crops Research Institute (CPCRI), Kasaragod, Kerala, India

**Keywords:** quercetin, bioactive compounds, antioxidant, antimicrobial properties, nutraceutical properties

## Abstract

Naturally occurring phytochemicals with promising biological properties are quercetin and its derivatives. Quercetin has been thoroughly studied for its antidiabetic, antibacterial, anti-inflammatory, anti-Alzheimer's, anti-arthritic, antioxidant, cardiovascular, and wound-healing properties. Anticancer activity of quercetin against cancer cell lines has also recently been revealed. The majority of the Western diet contains quercetin and its derivatives, therefore consuming them as part of a meal or as a food supplement may be sufficient for people to take advantage of their preventive effects. Bioavailability-based drug-delivery systems of quercetin have been heavily studied. Fruits, seeds, vegetables, bracken fern, coffee, tea, and other plants all contain quercetin, as do natural colors. One naturally occurring antioxidant is quercetin, whose anticancer effects have been discussed in detail. It has several properties that could make it an effective anti-cancer agent. Numerous researches have shown that quercetin plays a substantial part in the suppression of cancer cells in the breast, colon, prostate, ovary, endometrial, and lung tumors. The current study includes a concise explanation of quercetin's action mechanism and potential health applications.

## Introduction

The Latin term quercetum, which signifies Quercus robur (oak), is the source of the English word quercetin (1). Citrus-yellow quercetin crystals are soluble in lipids and alcohols but not in water (2). It has 15 carbon atoms and two aromatic rings connected by a 3-carbon bridge (3). Among the foods that contain this important nutritional flavanol are citrus fruits, flowers, green leafy vegetables, seeds, barks, buckwheat, broccoli, olives, almonds, onions, apples, red grapes, green tea, and berries. It is an aglycone that is present in the glycol-conjugates quercetin glucuronide, quercetin glycoside, and quercetin rutinoside, among others (4). Plants can be used to obtain quercetin. Compared to quercetin, the conjugated form of quercetin glycoside has a high absorption rate (5). About 77 times more quercetin is found in onion peel than in the edible section (6). Numerous methods, including solvent extraction, ultrasound-assisted extraction, microwave-assisted extraction, and the combination of strong pulsed light and subcritical water extraction, can be used to extract bioactive components from onion skin (6). For the first time, quercetin 3-galactoside has been found in the vegetative parts of *Azadirachta indica*, also known as neem. Its vegetative parts contain a variety of phytochemicals that may have biological and medicinal benefits, such as anti-cancer, anti-fungal, anti-bacterial, anti-viral, and anti-inflammatory properties (7). Quercetins have been used as dietary supplements because they provide protection against a variety of diseases, such as metabolic and inflammatory disorders. Additionally, it has immunomodulatory, anti-viral, anti-inflammatory, anti-cancer, anti-tumor, anti-allergy, anti-ulcer, and anti-cancer characteristics (8). One of the most significant endogenous antioxidants due to its protective nature against oxidants and inflammation, quercetin is widely used in pharmaceutical, cosmetic, and nutraceutical products (9). Quercetin has strong antioxidant, antibacterial, and anticancer properties, making it useful in a range of pharmaceutical and dietary applications (10). Despite having five hydroxyl groups, quercetin is lipophilic by nature, which results in a limited bioavailability. This disadvantage can be eliminated by encapsulating quercetin in a strong carrier that offers protection against oxidation, isomerization, and degradation, increasing its shelf stability and enabling its exaction and effective administration when swallowed (11). For instance, encapsulation in chitosan-coated zein nanoparticles can increase quercetin's bioavailability, chemical stability, intracellular antioxidant activity, and water-dispersibility (12). The oral absorption and bioavailability of quercetin in healthy subjects have reportedly been shown to suggestively increase when quercetin is consumed in a food-grade lecithin delivery system. This has reportedly been shown to effectively preserve physical conflict in triathletes and also demonstrate better control of allergic symptoms. During *in vitro* fecal incubation, it was discovered that native quercetin in the phytosome-formulated product was less susceptible to bacterial deterioration than it was in the unformulated one (13). Due to its ability to gel at room temperature, cold-set gel can also be used to encapsulate quercetin. Protein cold-set gels have been found to be more efficient than other hydrogels for protecting, displacing, and delivering heat-sensitive materials such vitamins, drugs, probiotics, and bioactive peptides. Therefore, protein cold-set gel can substitute for bioactive chemicals that are encapsulated and employed in functional food, cosmetics, pharmaceutical, and other industries. Free quercetin was found to have a 98 percent embedding rate and lower photochemical stability, thermal stability, and storage stability than quercetin embedded in porcine plasma protein (PPP) cold-set gel (14).

Many fruits and vegetables are resistant to Botrytis cinerea, blue mold, and other harmful plant infections because of quercetin. Therefore, there is a good likelihood that it will be employed as a natural manager for the postharvest preservation of fresh crops. On freshly cut fruit, active quercetin-chitosan (QCS) coatings can be applied to greatly reduce microbiological spoilage, moisture loss, and oxidative browning. Currently, QCS polysaccharide is utilized as an active ingredient in foods and other industries that require high levels of safety (15). Many macro and microminerals as well as nutrients like quercetin found in mangoes, including calcium (Ca), potassium (K), magnesium (Mg), manganese (Mn), phosphorus (P), iron (Fe), zinc (Zn), sodium (Na), copper (Cu), and selenium, are crucial for human health (Se) (16, 17). Additionally, it was noted that dried pineapple pulp had higher levels of quercetin, polyphenols, and flavonoids than fresh pineapple pulp (18). Both medicinal botanicals like *Ginkgo biloba* and a range of honey derived from different plant sources include quercetin. Due to changes in the intermolecular interactions between the hydrophilic groups of polylactide and polyethylene glycol and the polyhydroxyl groups of quercetin, it might be employed as an antibacterial agent while also improving the thermal and mechanical properties of polylactide-based films. Quercetin incorporation makes it possible to obtain antibacterial polymeric films (19). As quercetin's stability is typically impacted by oxygen concentration, concentrations of other antioxidants, pH value, temperature, and the presence of metal ions, chemical changes occur during processing and storage. Following ingestion, quercetin is absorbed in the intestines where it undergoes phase II metabolism and conjugates into methylated, sulfated, and glucuronidated derivatives in the blood. Thereafter, the kidneys and liver remove it in the form of bile, urine, and feces (4).

## Mechanism of action

**Figure 1:**
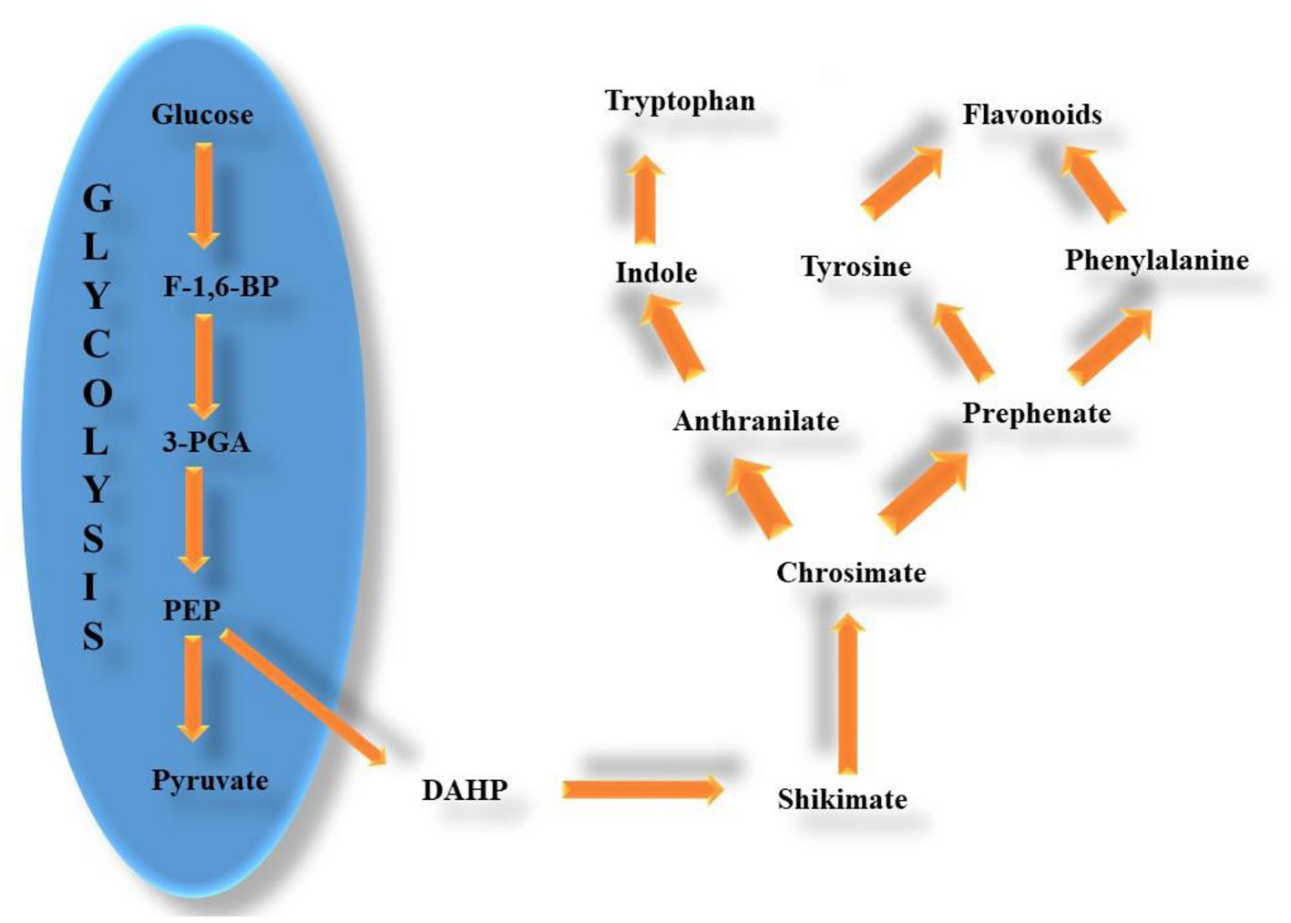
Pathway for synthesis of flavonoids in plants.

Quercetin, a natural antioxidant flavonoid provides protection mostly *via* the extinction of free radicals formed during cellular metabolism (20). Significant characteristics for quercetin antioxidant activity include dispersion in the membrane lipid bilayer, orientation, and affinity. The mechanism of action of quercetin has been attributed to its binding to or interference with enzymes, receptors, transporters, and signal transduction systems because quercetin (3,3^′^,4^′^,5,7-pentahydroxyflavone) is a polyphenolic component that possess broad biological activities primarily due to its antioxidant property. Given that the cell membrane concentrates the available quercetin molecules, investigating the biophysical characteristics connected to the variety of lipid compositions of cell membranes may be the key to understanding the involvement of cell membrane in these activities. These significant mechanisms primarily happen in membrane environments, inside, and through lipid bilayers (21). Figure 1 shows the pathway for the synthesis of flavonoids in plants.

## Potential health applications of quercetin

### Diabetes

Diabetes especially type II, is generally the predominant metabolic disorder occurring globally and is extremely diverse with fluctuating degrees of oxidative stress, insulin resistance and pancreatic β-cell dysfunction. In hyperglycemic conditions, increased fatty acid metabolism and glucose autoxidation produce surplus intracellular reactive oxygen species (ROS), which alter the structures and functions of the cells. This intensifies insulin resistance in insulin-sensitive cells and contributes to the development of diabetic complications (4). One of the factors contributing to the growth in diabetes incidence worldwide is the use of meals high in energy. According to estimates, diabetes and its complications cause more than 4.8 million deaths yearly (about 9 percent of the world's population). Lipid metabolism is impacted in the later stages of diabetes and manifests as hyperlipidemia and hypercholesterolemia, which are also risk factors for the disease. Changes in blood parameters are frequent among diabetics, indicating that the function of the blood cells is impaired since diabetes may have a detrimental impact on hematological parameters (22). By increasing the discharge of insulin and undeniably altering the unstable carbohydrate metabolic enzymes, it was discovered that the mixture of swertiamarin and quercetin (CSQ) had antihyperglycemic and antihyperlipidemic properties. Additionally, CSQ treatment demonstrated an impressive potential activity toward streptozotocin (STZ)-induced toxicity and safeguards the pancreas from oxidative stress-mediated hyperglycemia, which results in the renewal of the pancreatic islets and may increase exudation of insulin in STZ-induced diabetes (23). Additionally, it has been found that quercetin increases placental structural changes, like reduction in the thickness of the labyrinth interhemal membrane (LIM) and cell proliferation. It has also been found that by controlling adiponectin signaling and its receptors, quercetin can control these expression levels and restore them to nearly normal levels helps to prevent gestational diabetes mellitus as a result. Gestational diabetes mellitus (GDM), in general, is a metabolic disorder characterized by hyperglycemia brought on by abnormalities in insulin production, insulin action, or both, as well as increasing pregnancy-related issues. GDM is known to impact growth of fetus and function of placenta in humans and research animals, as well as raise intrauterine blood glucose levels (24).

### Hyperuricemia

Hyperuricemia has attracted increased attentiveness due to the health concerns of people because it is not only the primary cause of gout but is also a threat for developing other serious illnesses like cardiovascular disease (CVD) and chronic kidney disease (CKD). Basically, hyperuricemia is a state where the serum urate range is higher above the normal range, that is 2.5 to 7.0 mg/dl for men and 1.5 to 6.0 mg/dl for women. This elevated level results from either an excess of production from hepatic metabolism and cell turnover, an insufficient amount of excretion through the renal and digestive system, or a combination of these two factors (25). The last byproduct of purine metabolism, uric acid circulates in blood and is eventually eliminated by urine in humans and higher animals. The normal range of uric acid in blood is 360 and 420 μM in women and men, respectively, but close to neutral pH, such as in case of blood, concentrations >360 μM can initiate precipitation that leads to the accumulation of monosodium urate crystals in joint tissue interstitial spaces and gout (26). Increase in serum uric acid level causes weight gain thus leads to obesity which causes increase in uric acid synthesis and hinder its excretion *via* kidneys thus causing expansion in hyperuricemia (27). Liquid chromatography, bi-enzymatic colorimetry, capillary electrophoresis, and fluorescence spectroscopy are just a few of the systematic methods that have been developed to detect uric acid range in blood serum and urine of humans. But these methods have several limitations; they are expensive, require expensive equipment, and frequently produce inaccurate findings since they depend on physical characteristics, making them unsuitable for on-site uric acid monitoring. As a result, electrochemical sensors have drawn much greater attention due to their ease of use, affordability, high level of accuracy, and ability to do selective real-time uric acid measurement (28). Quercetin helps in prevention of hyperurecimia by showing

**Figure 2:**
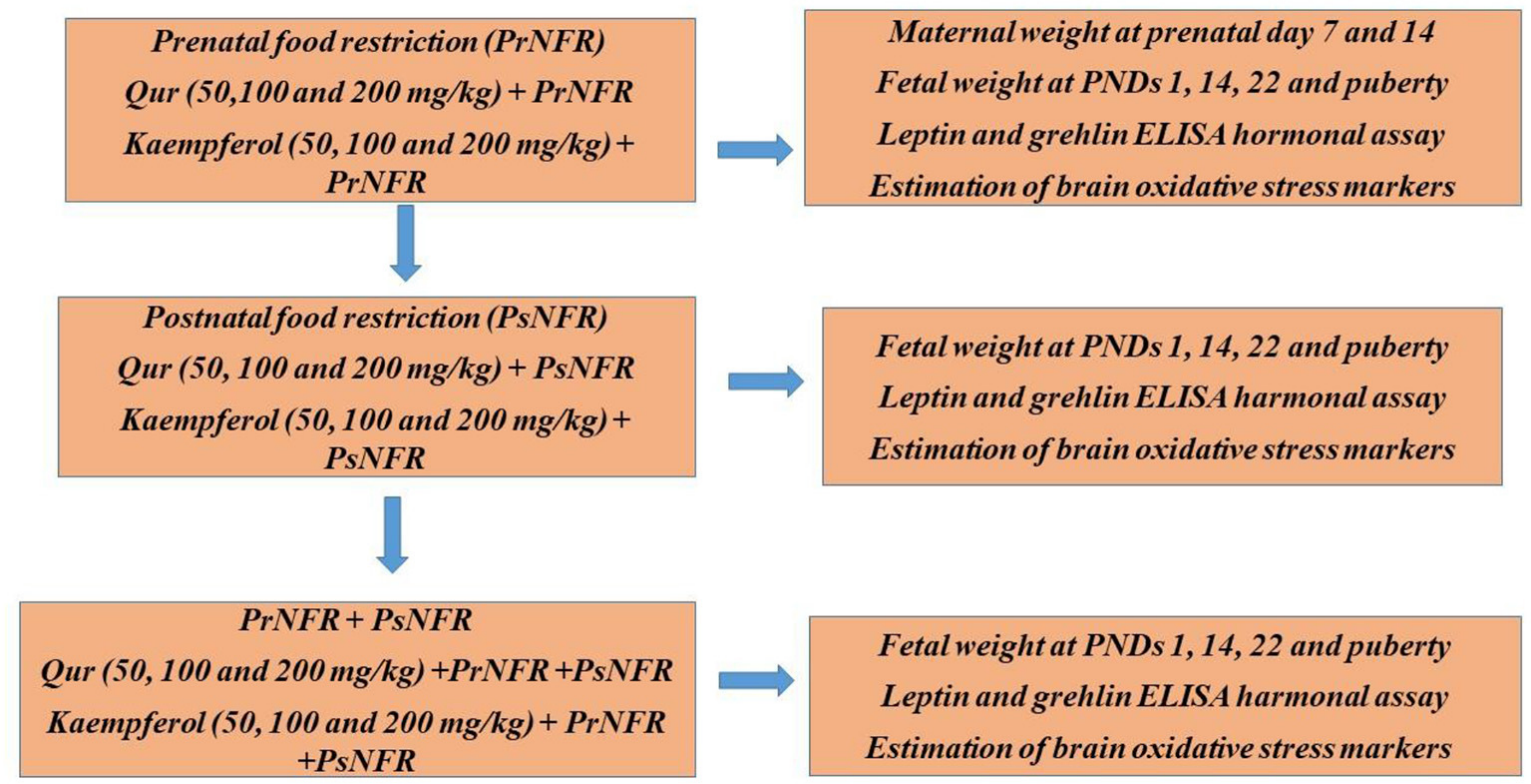
Experiment set up for pre and postnatal food deprivation.

**Figure 3:**
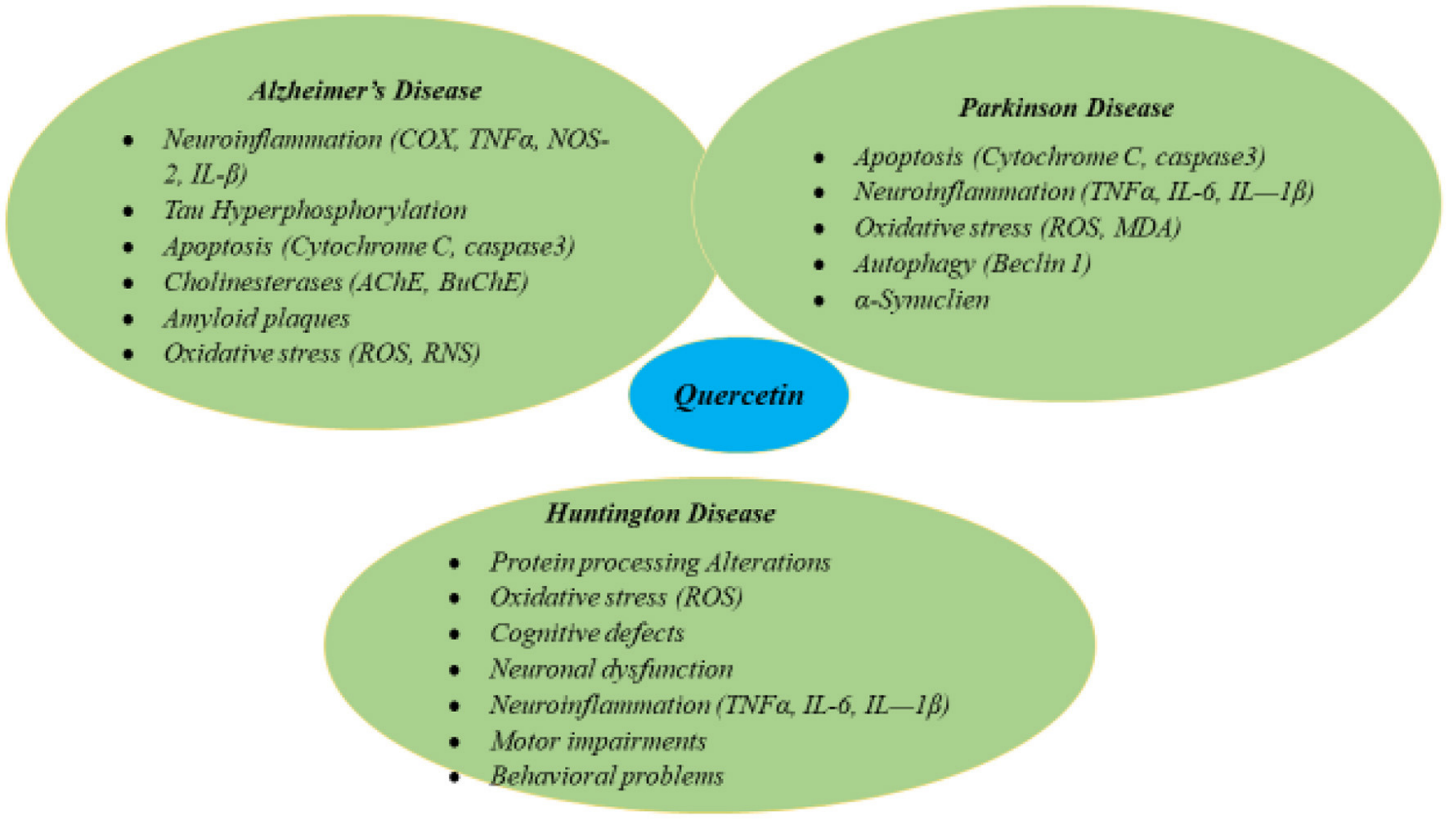
Molecular pathways involved in neuronal survival modulated *via* quercetin.

1) Urate production-related enzymes such adenosine deaminase, xanthine oxidase, and ketohexokinase are inhibited, and renal urate excretion is increased through supporting the activity of urate excretion transporters and subduing the activity of urate reabsorption transporters, among other methods (Figure 2).

2) Antioxidant activity by enhancing the antioxidant defense system and removing free radicals. Figure 3 depicts the various pathways modulated by quercetin.

3) Reducing gout and hyperuricemia-related comorbid conditions like hypertension, diabetes, obesity, dyslipidemia, CVD, and kidney disease.

As a result, it can be concluded that quercetin has the capability to function as a nutritional supplement in preventing and treating hyperuricemia and gouty arthritis. It can also be used in place of traditional medications or in conjunction with them to lessen their negative effects (25).

### COVID-19

Some naturopathic practitioners take supplements containing quercetin to support healthy immune function and reduce inflammation (29). Coronavirus disease 2019, also known as COVID-19, is a viral infection that affects lungs and is brought on by coronavirus-2 that causes severe acute respiratory syndrome. Globally, SARS-CoV-2 has been reported to have caused almost 240 million infected cases and almost 5 million fatalities. The therapeutic options include oxygen therapy and symptom management (30). Patients with non-severe COVID-19 typically experience signs and symptoms of mild unilateral or bilateral pneumonia, including fever, tiredness, dry cough, sputum production, headaches, and dyspnea. However, the primary causes of death in individuals with severe COVID-19 disease are pneumonia, acute respiratory distress syndrome (ARDS), metabolic acidosis, thromboembolism, multiple organ failure, and sudden heart damage. Different legal medications are being used for COVID-19 treatment based on information from a mechanistic knowledge of COVID-19 etiology and big-data analysis employing artificial intelligence. The immune system has responded favorably to numerous doses of the SARS-CoV-2 vaccine. The most effective method of avoiding transmission is mass immunization, although it is premature to presume that the first generation of the vaccine will be successful in preventing COVID-19 infection and transmission (31). The World Health Organization-synchronized medications used in the world's largest COVID-19 randomized control trial deceitfully showed to have slight or no effect on covid patients who were hospitalized. This has rekindled research into alternative repurposed medications or efficient “add-on” dietary supplements that could enhance or supplement the therapeutic effect of repurposed medications. It's interesting to note that the natural flavonoid quercetin has the ability to lower blood cholesterol levels in macrophages *via* altering the expression of ABCA1, which is a key regulator of reverse cholesterol transport. By enlightening the dysregulated cholesterol metabolism and persistent inflammation during the initial stages of atherosclerosis, quercetin treatment reduced the production of foam cells (32). Angiotensin-converting enzyme II (ACE2) has been found to be the human SARS-CoV-2 receptor, and since ACE2 is required for SARS-CoV-2 cell entrance, an ACE2-targeting method holds tremendous promise for the development of drugs to combat COVID-19. Surprisingly, quercetin was able to attach to the S-receptor-binding protein's domain (RBD), indicating that it not only blocked receptors but also rendered SARS-CoV-2 virus ineffective (33) (Table 1). Quercetin is a potential COVID-19 candidate since the results of network pharmacology and bioinformatics analysis support the notion that it takes part in host immunomodulation (41).

**Table 1:** The inhibitory effect of quercetin on bacteria.

**Bacteria**	**Mechanism of action**	**Reference**
*Escherichia coli*	Inhibition of nucleic acid formation and disruption of plasma membrane function	(34–36)
*Salmonella typhimurium*	Inhibition of nucleic acid formation and disruption of plasma membrane function	(34)
*Bacillus subtilis*	Destruction of bacterial cell walls and cell membranes	(37)
*Pseudomonas aeruginosa*	Inhibition of nucleic acid synthesis and disruption of plasma membrane function	(34, 35)
*Streptococcus mutans*	Reduction of dry weight of biofilm and total proteins	(38)
*Mycobacterium*	Inhibition of glutamine synthetase by Quercetin-3-O-β-D-glucoside	(39)
*Enterococcus faecalis*	Inhibition of synthesis of *Enterococcus faecalis* naphthalatesynthas	(40)

### Bone formation

The restricted availability of bone and susceptibility to donor site difficulties make the use of autologous bone transplantation extremely difficult for bone abnormalities that are typically brought on by trauma, tumors, and infection. Therefore, it is critically necessary to improve artificial bone replacement materials with superior biocompatibility, bone conductivity, and bone inductivity, for restoring bone flaws. The strong osteo-inductive and angiogenic capabilities of quercetin and its derivatives may support the development of bone marrow stem cells (BMSCs) into osteoblasts (42). Table 2 contains an organized information about amount obtained from various methods. The aging of the global population, which increases the risk of musculoskeletal and dental illnesses such bone resorption, bone fractures, and bone tumors or infections, has led to a surge in bone grafting treatments over the past few decades. The calcium silicate-based materials have been the most frequently used among different bone graft materials to strengthen and regenerate hard tissue because they tempt the formation of bone-like apatite by steadily releasing silicon ions both *in vitro* and *in vivo*, which stimulate the expression of proteins related to bone and regulate the proliferation and differentiation of mesenchymal stem cells and pulp stem cells. Therefore, it is acknowledged that the calcium silicate-based ceramic plays a vital role in bone regeneration, especially in the healing and reconstruction of hard tissues. However, calcium silicate has been discovered to have vital draw backs in terms of therapeutic purposes, meaning that it can't be destroyed in a timely manner. The tissue around the bone graft doesn't have the ability to effectively migrate toward the interior of the substance once it has become anchored in the body. As a result, the use of calcium silicate substances in tissue engineering and innovative medicine is hindered. It has been demonstrated that quercetin positively interacts with bone cells, promoting osteoblast growth, and effectively slows or stops osteoclast activity. According to research, quercetin plays a key role in regulating the processes of bone tissue renewal and inflammation (6). Quercetin aids in treating diabetic osteopenia as it lowers blood glucose levels. Diabetes mellitus and osteopenia are intimately related, increasing the risk of bone fractures and delaying the healing of fractures. Compared to quercetin in its free form, derivatives of quercetin appear to have stronger bone-protective properties (49). In estrogen deficiency-induced bone loss and ovariectomized rats, quercetin and kaempferol prominently advance bone mineralization, bone microstructure, and osteoblast activity (50). Table 3 includes different health benefits of quercetin. The chronic inflammatory disease rheumatoid arthritis (RA), which is marked by synovial inflammation and joint destruction, is another condition for which quercetin is crucial in prevention. Bone metabolism is significantly impacted by increased quantity of tumor necrosis factor-a (TNF-a), enhanced oxidative stress, interleukin (IL)-6, and IL-1b. Reactive oxygen species (ROS) cause osteoclast differentiation and bone resorption, whereas oxidative stress and inflammatory cytokines stimulate osteoclast development. Osteoclasts are specialized bone-resorbing cells that are regulated by nuclear factor kappa-B ligand and macrophage colony-stimulating factor (M-CSF) (RANKL). Quercetin has demonstrated effectiveness in treating RA. Thus, it can be inferred that quercetin-controlled osteoclast genesis in RA in a variety of ways, including by reducing the level of RANKL in RA-FLS, inhibiting the osteoclast differentiation of monocytes from RANKL and IL-17, and regulating the development of Th17 cells (6).

**Table 2:** Amount of quercetin obtained by different methods.

**Extraction method**	**Quantity**	**Reference**
Intense pulsed light and subcritical-water extraction	17.32 ± 1.12 mg/g onion skin at 145°C for 15 min	(43)
Pulsed electric field and subcritical-water extraction	Yield improved by 33.22% and decrease in temperature from 165 to 145°C	(44)
Methanol extraction	101.4 ± 1.1, QE/g dry extract	(45)
Ethanol extraction	120.6 ± 1.3, QE/g dry extract	(45)
Ultrasound assisted extraction	0.29 QE mg/g	(46)
Microwave assisted extraction	35.9 QE mg/g	(47)
Enzyme-assisted extraction	12.56 QE μg/g	(48)

QE, Quercitrin equivalent.

**Table 3:** Health benefits and mechanism of quercetin.

**Property**	**Mechanism**	**Reference**
Diabetes	Reduction in the thickness of the labyrinth interhemal membrane (LIM) and cell proliferation thus helps in prevention of gestational diabetes mellitus	(24)
Hyperuricemia	Increases renal urate excretion, free radical scavenging	(25)
COVID-19	Displays the anti-hypercholesterolemic property and reduce the deposition of cholesterol in macrophages	(32)
Bone formation	Inhibits osteoclast activity	(6)
Neuroprotection	Reactive oxygen scavenging	(51)
Tuberculosis	Acts on isocitrate lyase, an enzyme necessary for attaching the *M. tuberculosis* to host cells	(52)
Cardio protection	Inhibits apoptosis and reduces oxidative stress and inflammatory proteins in the heart	(53)
Cancer	Inhibits cell proliferation	(54)

### Neuroprotective agent

A natural flavonoid called quercetin has neuroprotective properties in addition to acting as an antioxidant and effective metal chelator. In addition, quercetin increases toxicity-induced neuronal cell damage and stabilizes intracellular calcium concentration. Ischemic stroke causes neuronal cell death due to an unnecessary intracellular calcium excess. But in ischemia affront and oxidative stress models, quercetin reduces the calcium excess. It is likely that the calcium-binding protein is modulated in quercetin's neuroprotective activity against cerebral ischemia (55). Quercetin and kaempferol have been shown in numerous papers to attenuate various metabolic syndromes linked to prenatal and early postnatal stress through pathways involving suppression of oxidative stress and inflammatory process in entire animals and cultured human endothelia cells. Overall, the research by Anachuna et al. (50) acknowledged potential links between prenatal and early postnatal food limitations, altered orexigenic/anorexigenic hormone levels in the serum, and elevated brain oxidative stress in Wistar rats, which is reversible by antioxidant therapies. The effects of prenatal, postnatal, or combination food shortages on rats' pup weights were found to be enhanced by quercetin and kaempferol through mechanisms including the modulation of leptin and ghrelin levels including antioxidant pathways. In order to combat alterations in metabolic and brain functioning brought on by prenatal and early postnatal dietary shortage, quercetin and kaempferol supplementation may be essential (50).

Quercetin plays a significant part to prevent oxidative stress, mitochondrial dysfunction, neuroinflammation and apoptosis that are linked to neurodegenerative diseases (NDDs). NDDs are actually heterogeneous disorder are categorized by the regular progressive neuronal loss. These are set of late-onset, advanced, age-dependent brain disorders, symbolized medically by the decreased control of cerebra, dyskinetic movements motor activity, and perpetual changes in behavior and nature. Pathological marks of this condition including Parkinson's disease (PD), Alzheimer's disease (AD) and Huntington's disease (HD), are deposits of mutant proteins like α-synuclein, amyloidβ (Aβ) and mutant huntingtin (Htt), respectively in the affected regions of brain. AD is an enduring NDD that causes substantial withering of the frontal cortex, cingulate gyrus, temporal, and parietal lobes as well as the loss of neurons and synapses in the cerebral cortex and some subcortical areas. The presence of neurofibrillary tangles (NFTs) and senile plagues serve as indicators of the AD disease (51).

Quercetin was advertised as having the ability to reduce oxidative stress and neurotoxicity when administered *in vivo* and lured by a variety of broadsides, including metals, pesticides, and neurotoxins. In rat models of intracerebral hemorrhage, transgenic mouse models of AD, and streptozotocin-induced diabetic rat models, quercetin also improved neurological diseases. However, quercetin treatment of healthy animals also produced adverse effects on the neurological system. It was discovered that while quercetin exposure alone had negative effects on fish (*Channa punctata*), it had favorable or protective effects on fish with oxidative stress brought on by prior deltamethrin exposure. Depending on the redox state, quercetin had a good or detrimental effect on mice's cognitive function. Thus, it may be inferred that low levels of quercetin may benefit the neurological system whereas large levels may have adverse consequences (56).

### Prevention of tuberculosis

**Figure 4:**
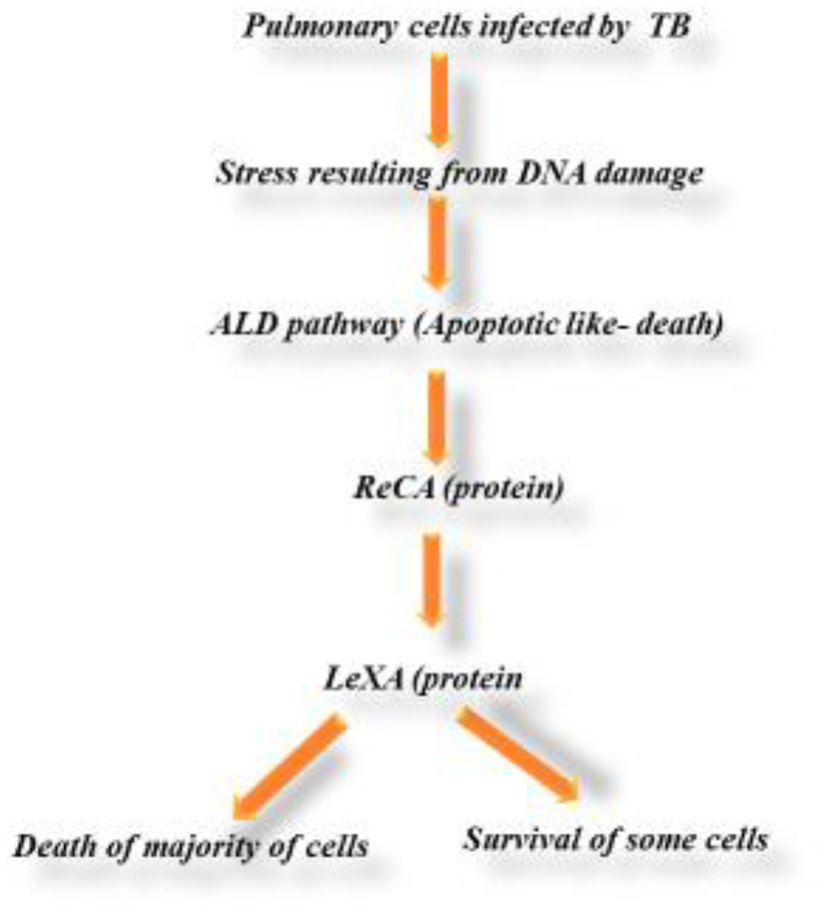
Mechanism of DNA damage in tuberculosis (TB).

The random utilization of commercial or synthetic antimicrobial medications, that are typically used to treat various infectious diseases, has prompt the development of Multiple Drug Resistance (MDR) in human pathogenic microorganisms. This circumstance is ideal for creating novel antibacterial substances from different natural sources. Synthetic medications possess severe impact on human health and are also quite expensive to produce. Traditional medicines, especially plants, have become a major force in the field of medicine since they are widely available and almost always free from adverse effects (57). Tuberculosis is a transmittable disease and its causative agent is *Mycobacterium tuberculosis*. It is the main source of mortality infection. Universally, it was estimated that 10 million people are affected by this disease with 1.41 million deaths in 2019. Thus, the administration of numerous medications over an extended period of time is necessary for the treatment of tuberculosis. Whereas, the typical regimen included isoniazid, rifampicin, pyrazinamide, and ethambutol dihydrochloride (EDH) for about 2 months, followed by four more months of isoniazid and rifampicin. Unfortunately, multidrug-resistant (MDR) tuberculosis control is waning while therapy has barely advanced (58). ROS-mediated oxidative damage is considered to be the primary cause of lipid peroxidation and subsequently the hepatic injury, even if the mechanism and causative elements of hepatotoxicity caused by anti-tuberculosis drugs remain unclear. Anti-TB medications have been shown to alter both enzymatic and non-enzymatic components of the cell defense mechanism (59). The peculiar character of *M. tuberculosis* is its cell wall containing good profile of lipids, helping the bacteria in maintaining its intracellular survival and pathogenicity. The cell wall synthesis pathway is a promising mark for new anti-TB drug discovery. The enzyme pantothenate synthetase (PS or PanC), is a key enzyme essential for the biogenesis of coenzyme A (CoA). It produces pantothenate (vitamin B5), which is pioneer for the biosynthesis of CoA. The CoA is a crucial element of fatty acid synthesis. If this enzyme is repressed, the fatty acid synthesis of *M. tuberculosis* will be disturbed which then will affect the synthesis of cell wall (60). Quercetin's growth-inhibitory properties against *M. tuberculosis* H37Rv were highly effective and notable. Quercetin's ability to fight tuberculosis (TB) is a sign of how well-suited it is to serve as a prototype for future anti-TB drug delivery methods. Furthermore, there is ample documentation that the patients with destructive pulmonary tuberculosis (DPTB) who are receiving chemotherapy in combination with quercetin-fixed polyvinylpyrrolidone (PVP) have an improved prognosis. When coupled with PVP, quercetin functions as an antioxidant, a capillary stabilizer, and may have immunomodulatory effects (61). Through the release of bacilli from an infected person's lungs into the surrounding atmosphere, TB is transmitted. These microbes are then gulped by vulnerable individuals–especially those having close interaction and immunocompromised individuals. Then, as these organisms increase in the lungs, the host's immune system is stimulated, especially the activation of phagocytes. Granuloma, a mass of necrotic lung tissue and immune cells such T cells, B cells, and macrophages, is the name given to this outcome. The pathologic representation of the host's reaction to TB infection is this granuloma. People with active TB have symptoms include decreased appetite, fever, lethargy, and weight loss. Those with advanced pulmonary disease frequently cough up blood while those with lung disease frequently have a persistent cough (haemoptysis). Isocitrate lyase, an enzyme required for the attachment of *M. tuberculosis* to host cells, was the target of quercetin. Tricarboxylic acid (TCA) cycle functionality and cyclic cell development depend on isocitrate lyase. Quercetin improves the repressive effect on *M. tuberculosis* metabolism by lowering *M. tuberculosis* isocitrate lyase (52). It was discovered that quercetin suppressed 99.30 0.268 percent of the luciferase reporter phase a assay against *M. tuberculosis* at a dose of 200 g/ml. This phenolic compound's claimed ability to inhibit M. tuberculosis glutamate racemase (Murl), a component of cell wall peptidoglycan (PG) formation, was confirmed by an *in silico* research (62) (Figure 4).

### Cardioprotective agent

**Figure 5:**
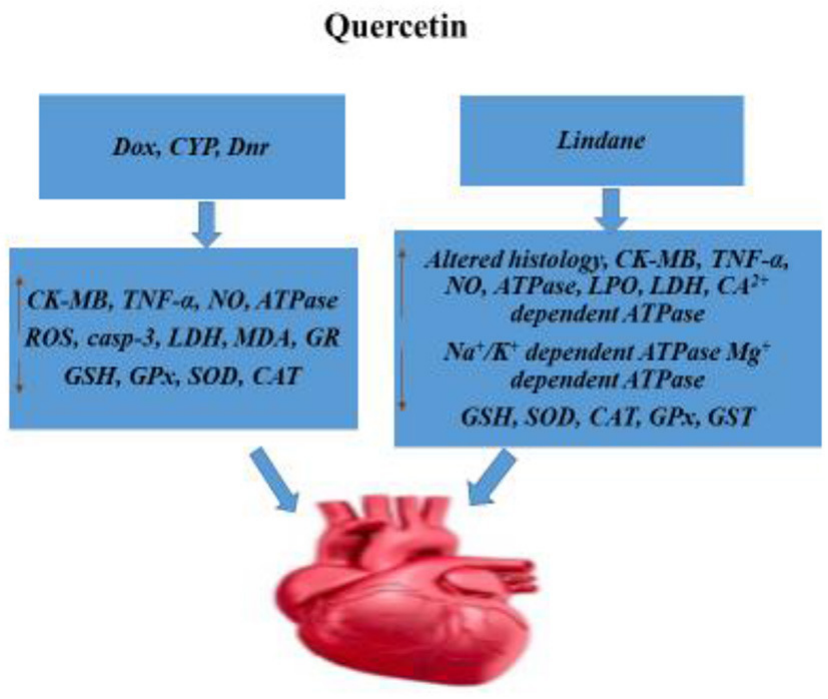
Cardiac damage (cardiotoxicity). Schematic representation of the protective mechanisms of quercetin to mitigate cardiac toxicity. Dox, Doxorubicin; CYP, Cyclophosphamide; Dnr, Daunorubicin; CK-MB, Creatine kinase-MB; TNF-α, Tumor Necrosis Factor-alpha; NO, Nitric Oxide; ROS, Reactive Oxygen Species; Casp-3, Caspase-3; LDH, Lactate Dehydrogenase; MDA, Malondialdehyde; GR, Glutathione reductase; GSH, Glutathione; GPx, Glutathione Peroxidase; SOD, Superoxide dismutase; CAT, Catalase; LPO, Lipid Peroxidation; GST, Glutathione S-transferase.

17.0 million individuals globally every year, or 31% of all mortality, suffer from cardiovascular disease (CVD). By 2030, this number is expected to reach 25 million. Important discoveries indicate that hypoxic environments promote oxidative stress, which is thought to be a major contributor to CVD. The majority of the time, hypoxia is detrimental to the heart's ability to operate because, in response to these stimuli, cardiomyocytes undergo apoptosis, which could result in a loss of function. This disruption of heart function results in significant and long-lasting harm to the entire human body. An efficient preventive or therapeutic approach for CVD is to stop the progression of apoptosis because of the critical role that cardiomyocyte omission plays in morbidity and death (63). Quercetin extracted from fruits and vegetables has the wonderful potential to function as a cardioprotective agent, according to pharmacological hypotheses. The antioxidative and antiplatelet properties of the muscle have a significant impact on its function. These entities engage in behaviors including inhibiting smooth muscle cell migration and proliferation, which improves the mitochondrial function of cardiac cells and inhibits nuclear factor-kappa light chain-enhancer of activated B cells (NF-B) (64). By reducing oxidative stress, educating the renin-angiotensin-aldosterone system (RAAS), and enhancing vascular function, quercetin is thought to reduce blood pressure. When quercetin is taken by people with hypertension or pre-hypertension at doses of 500 mg/day and above, it has a significant anti-hypertensive impact. Nitric oxide produced by the endothelium may be more bioavailable due to quercetin, which would improve vascular function. Quercetin doses above 50 mg/day were said to suggestively lower blood triglyceride levels while having no discernible effect on other plasma lipids. Quercetin plays a key role in lowering cardiovascular risk factors like fibrinogen and C-reactive protein because of its anti-inflammatory effects (65). Atherosclerosis (AS) is a chronic, inflammatory, and inflammatory disease that is characterized by inflammation, endothelial dysfunction, abnormalities in lipid metabolism, migration and proliferation of smooth muscle cells, and oxidative stress. With a mortality rate of more than 40% and an increasing incidence and case count, atherosclerotic cardiovascular disease (ASCVD), which is brought on by AS, is one of the conditions with the greatest rates of morbidity and mortality worldwide. It also ranks first among all causes of death among urban and rural Chinese residents. Through its anti-inflammatory, antioxidative, regulation of lipid metabolism abnormalities, and other pharmacological activities, quercetin can diminish the formation of AS plaque in the treatment of ASCVD, lowering the prevalence of ASCVD and taming the diagnosis of ASCVD patients (66). In females, estrogen deficiency raises the risk of depression. Estrogen primarily exerts its biological effects by activating its receptors, estrogen receptor (Er) and ER, intracellularly. Additionally, the formation of the estrogen-ER complex can indicate the transcription of thousands of heritable factors. The sensitivity of different tissues to estrogen among biological components may aid in explaining the symptom variability, bone loss, and the risk of CVD and depression. Epidemiological research has established a direct relationship between depression and heart disease. The potential association between depression and cardiovascular illness and brain-derived neurotrophic factor (BDNF) has also been confirmed by recent research. A member of the neurotrophic factor family, BDNF controls the growth, maintenance, and survival of neurons. It was first discovered in brain tissue. By lowering hippocampus oxidative stress, QUE is known to lessen the behavioral dysfunction that depression-like stress causes (67). Atherogenesis, which causes myocardial infarction, stroke, and diabetic foot ulcers followed by gangrene, is accelerated by cardiovascular issues. Cardiomyopathy in diabetics. In addition to cardiomyopathy, hypertrophy, congestive heart failure, heart valve disease, and ischemic heart disease, free radicals have a role in the etiology of numerous cardiovascular disorders (68). In addition to its vascular impact, quercetin exhibits strong heart protection against a variety of cardiac injuries, such as ischemia-reperfusion (I/R) injury, doxorubicin-induced cardiotoxicity, diabetic cardiomyopathy, and others. It has cardioprotective effects *via* regulating a variety of signaling pathways and proteins, such as decreasing oxidative stress and inhibiting apoptosis, as well as by affecting inflammatory proteins in the heart (53) (Figure 5).

### Anti-cancer agent

**Figure 6:**
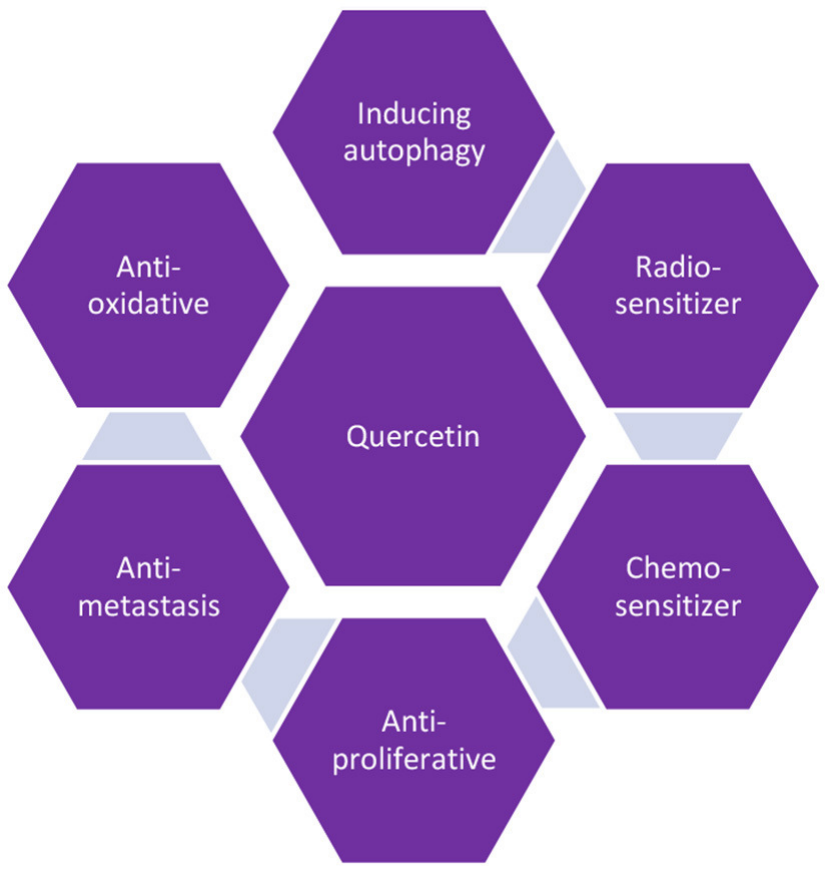
Quercetin beneficial roles in cancer treatment.

The problem of cancer is having global significance and is the second important reason of deaths around the globe. This pathology has a great influence on economy of public and private health. For example, in 2010, the annual economic cost of cancer in the US was $1.16 trillion USD. According to a prediction from Cancer Tomorrow, there would be a 63.1 percent increase in new cases of cancer overall in 2040. Lung (11.6 percent of all cases), breast (11.6 percent), colorectal (10.2 percent), prostate (7.1 percent), and stomach cancer are the cancers that occur most frequently globally (5.7 percent). Lung cancer (18.4%), colorectal cancer (9.2%), stomach cancer (8.2%), liver cancer (8.2%), and breast cancer are the top causes of death worldwide. Although high incidence rates are associated with high-income countries, low- and middle-income countries account for 70% of all deaths (69). Cancer is a form of disease with characteristics similar to other diseases, not a recently recognized ailment with a particular cause. According to earlier research, the control of proliferative factors, evasion of growth inhibitory factors, suppression of cell death, replicative immortality, angiogenesis, and increased invasion and metastasis are all indications of cancer. These six biological processes are supported by genetic variety brought on by genomic instability. Surgery, radiation, chemotherapy, and immunotherapy are frequently used singly or in combination as cancer treatments. The key factor impeding chemotherapy medications' efficiency is resistance to drugs as cancer cells grow used to chemotherapeutic agents, which occurs concurrently with their use. The need for the creation of substitute medications results from this (5). Since ancient times, plants have been utilized to cure a variety of infectious diseases; some of these plants are being used today as standard therapies for many ailments. Since the previous age, both in industrialized and emerging nations, there has been a significant increase in the use of and public interest in natural rehabilitation. The availability of these herbal medications has expanded to include grocery and supermarket stores in addition to drug stores. Due to their accessibility and affordability compared to synthetic compounds, traditional herbal treatments are still used to treat ailments by almost 80% of the population in Africa and other impoverished countries. These natural substances also have anti-inflammatory, spasmolytic, antioxidant, sedative, antimicrobial, antiviral, antiseptic, anti-diabetic, immunostimulant, and hepatoprotective activities. They posses great potency to treat a variety of medical conditions, including respiratory and gastrointestinal disorders (70). Researchers in the field of drug discovery are paying close attention to traditional treatments for cancer. On the other hand, recognized chemotherapeutics are improved by using naturally generated substances as adjuvant agents. The necessary and promising chemotherapeutic properties as well as chemomodulatory anticancer effects were demonstrated by a variety of chemical families and bioactive substances of plant origin. Various fruits and vegetables contain the polyphenolic chemical quercetin, which has antioxidant, anti-inflammatory, and antibacterial characteristics (71) (Figure 6).

In the United States, colon cancer is the third most frequent cancer in both men and women and the third leading cause of cancer-related mortality (Cancer country profiles 2014, WHO). The likelihood of developing colon cancer appears to be firmly correlated with dietary practices, and the western-style diet is thought to raise the risk. According to information published by the WHO, lifestyle habits, such as food, exercise, or use of alcohol and cigarettes, stoutly promote the occurrence of cancer. In their work, Langner et al. established that the combination of the naturally occurring phytochemicals lycopene, sulforaphane, quercetin, and curcumin reduced the growth of colon cancer cells while having no negative effects on healthy colon epithelial cells. They got to the conclusion that the suggested mixture of phytochemicals with empirically shown antiproliferative activity may be useful as a proactive strategy for the prevention and treatment of colon cancer (54). The epithelial layer of the colon and rectum tissue is where colorectal cancer (CRC) first develops. The fourth most typical cause of cancer-related fatalities worldwide is CRC. Because of the western lifestyle, the rate of CRC has risen over the past 10 years in both high-income and developing nations, and is expected to continue to rise in the years to come. Depending on the stage of the tumor, traditional treatment for CRC may vary, although it typically entails standard radiation and chemotherapy. Due to their limited effectiveness and numerous adverse effects, including low immunological response, hair loss, fatigue, nausea, and hormone imbalance. Therefore, it is essential to create novel therapeutic strategies for CRC therapy and prevention (72). Various researches establish the disruption of the intestinal microbial ecology as a cause of colorectal cancer (CRC). Quercetin-2,3-dioxygenase, also known as quercetinase, has been identified as the first enzyme in Bacillus spp. necessary for the metabolism of quercetin. The cleavage of quercetin by this enzyme is accompanied by the action of gut esterases on the intermediate product [2-(3,4-dihydroxybenzoyloxy)-4,6-dihydroxybenzoate], which can result in the production of 2,4,6-trihydroxybenzoic acid (2,4,6-THBA) and 3,4-dihydroxybenzoic acid (3,4-DHBA), both of which are known to have antiproliferative properties in cancel cell (73). Breast cancer (BC) is regarded as a rising female delinquent in recent years. Because of the wide diversity of lifestyles created, it is one of the main reasons for death. Cancer prevention and therapy using natural dietary components has gained current medical attention. Because of its pro-oxidant properties, quercetin is used to control tumor growth. Additionally, it triggers the onset of apoptosis and arrests the cell cycle (74).

Ovarian cancer is ranked as the sixth most general disease in women globally, with ~240,000 new cases, according to a 2018 report. As ovarian cancer is linked with silent and ambiguous symptoms, it is typically discovered in advanced stages. Despite much data being gathered on this obstacle recently, the survival rate has not improved considerably because to various difficulties in accurately detecting and treating it. Launching a treatment platform for ovarian cancer is difficult due to various changes at the molecular and genetic levels (75). Abdominal pain, bloating, frequent urination, and changes in bowel habits are a few symptoms of ovarian cancer. It is crucial that healthcare organizations take these hazy and vague symptoms into account, especially in high-risk instances. Ovulation, endometriosis, nutritional factors, race, and family history or genetic susceptibility are among the risk factors for this disease. The OH group at C3 location and the presence of catechol in quercetin provide an excellent arrangement for scavenging free radicals. Due to its potential for working in concert with other chemo-preventive medications, quercetin is a potential option for the treatment of ovarian cancer (76).

## Conclusion

Natural bioactive compounds are receiving a lot of attention, and quercetin is one of the best examples of how they may be used in amazing medicinal ways. Apples, onions, and berries are among the foods known to contain quercetin, a flavonoid having antioxidant qualities. Quercetin has demonstrated promising therapeutic effects against a number of illnesses, including lung cancer, cardiovascular disease, and osteoporosis. It has been demonstrated that those who consume a lot of flavonoids have a lower chance of developing cardiovascular disease. Clinical applications for its potent antioxidant, anti-inflammatory, and anti-tumor properties are very promising. The antibacterial activity of quercetin, which has significant implications for the security and long-term improvement of human and animal health, is currently the subject of scant investigation. However, quercetin's broad-spectrum antibacterial characteristics can be utilized to treat a range of infectious bacterial infections, both in terms of prevention and treatment, and as a substitute for antibiotics. Further experimental study is required to determine whether quercetin has inhibitory effects on various types of fungi or whether its antibacterial mechanism is comparable to that of fungi and bacteria.

## Author contributions

All authors listed have made a substantial, direct, and intellectual contribution to the work and approved it for publication.

## Funding

This work was supported by a grant from the Romanian National Authority for Scientific Research and Innovation, CNCS—UEFISCDI, project number PN-III-P2-2.1-PED-2019-1723 and PFE 14, within PNCDI III. The publication of this article was funded by the Open Access Fund of the Leibniz Universitt Hannover, Germany.

## Conflict of interest

The authors declare that the research was conducted in the absence of any commercial or financial relationships that could be construed as a potential conflict of interest.

## Publisher's note

All claims expressed in this article are solely those of the authors and do not necessarily represent those of their affiliated organizations, or those of the publisher, the editors and the reviewers. Any product that may be evaluated in this article, or claim that may be made by its manufacturer, is not guaranteed or endorsed by the publisher.

## References

[1] Singh P Arif Y Bajguz A Hayat S. The role of quercetin in plants. Plant Physiol Biochem. (2021) 166:10–9. 10.1016/j.plaphy.2021.05.023 34087741

[2] Crnivec IGO Skrt M Šeremet D Sterniša M Farčnik D Štrumbelj E. Waste streams in onion production: bioactive compounds, quercetin and use of antimicrobial and antioxidative properties. Waste Manag. (2021) 126:476–86. 10.1016/j.wasman.2021.03.033 33838387

[3] Lin J Zhou W. Role of quercetin in the physicochemical properties, antioxidant and antiglycation activities of bread. J Funct Foods. (2018) 40:299–306. 10.1016/j.jff.2017.11.018

[4] Dhanya R. Quercetin for managing type 2 diabetes and its complications, an insight into multitarget therapy. Biomed Pharmacother. (2022) 146:112560. 10.1016/j.biopha.2021.112560 34953390

[5] Tang SM Deng XT Zhou J Li QP Ge XX Miao L. Pharmacological basis and new insights of quercetin action in respect to its anti-cancer effects. Biomed Pharmacother. (2020) 121:109604. 10.1016/j.biopha.2019.109604 31733570

[6] Huang KH Chen CY Chang CY Chen YW Lin CP. The synergistic effects of quercetin-containing 3D-printed mesoporous calcium silicate/calcium sulfate/poly-ε-caprolactone scaffolds for the promotion of osteogenesis in mesenchymal stem cells. J Formos Med Assoc. (2021) 120:1627–34. 10.1016/j.jfma.2021.01.024 33593691

[7] Rao PS Subramanayam G Sridhar PR. Quercetin 3-galactoside from *Azadirachta indica*. J Adv Mol Biol. (2019) 3:1–8. 10.22606/jamb.2019.31001

[8] Janeczko M Gmur D Kochanowicz E Górka K Skrzypek T. Inhibitory effect of a combination of baicalein and quercetin flavonoids against Candida albicans strains isolated from the female reproductive system. Fungal Biol. (2022) 126:407–20. 10.1016/j.funbio.2022.05.002 35667828

[9] Wadhwa K Kadian V Puri V Bhardwaj BY Sharma A Pahwa R. New insights into quercetin nanoformulations for topical delivery. Phytomedicine Plus. (2022) 2:100257. 10.1016/j.phyplu.2022.100257

[10] Ezati P Rhim JW. Fabrication of quercetin-loaded biopolymer films as functional packaging materials. ACS Applied Polymer Materials. (2021) 3:2131–7. 10.1021/acsapm.1c00177

[11] Ghatak D Iyyaswami R. Selective encapsulation of quercetin from dry onion peel crude extract in reassembled casein particles. Food Bioprod Process. (2019) 115:100–9. 10.1016/j.fbp.2019.03.003

[12] Zou Y Qian Y Rong X Cao K McClements DJ Hu K. Encapsulation of quercetin in biopolymer-coated zein nanoparticles: formation, stability, antioxidant capacity, and bioaccessibility. Food Hydrocolloids. (2021) 120:106980. 10.1016/j.foodhyd.2021.106980

[13] Di Pede G Bresciani L Calani L Petrangolini G Riva A Allegrini P. The human microbial metabolism of quercetin in different formulations: an *in vitro* evaluation. Foods. (2020) 9:1121. 10.3390/foods9081121 32823976 PMC7466208

[14] Yuan JL Ding CS Li CL Zhang Y Kang X. Protective, controlled-release and embedding mechanism of porcine plasma protein cold-set gel on quercetin: an effective carrier of hydrophobic compounds. Food Biosci. (2022) 47:101672. 10.1016/j.fbio.2022.101672

[15] Shebis Y Laskavy A Molad-Filossof A Arnon-Rips H Natan-Warhaftig M Jacobi G. Non-radical synthesis of chitosan-quercetin polysaccharide: properties, bioactivity and applications. Carbohydr Polym. (2022) 284:119206. 10.1016/j.carbpol.2022.119206 35287917

[16] Sarkar T Salauddin M Sheikh HI Pati S Chakraborty R. Effect of drying on vitamin, carotene, organic acid, mineral composition, and microstructural properties of mango (*Mangifera indica*). Journal of Food Processing and Preservation. (2022) 46:e16237. 10.1111/jfpp.16237

[17] Sarkar T Bharadwaj KK Salauddin M Pati S Chakraborty R. Phytochemical characterization, antioxidant, anti-inflammatory, anti-diabetic properties, molecular docking, pharmacokinetic profiling, and network pharmacology analysis of the major phytoconstituents of raw and differently dried mangifera indica (himsagar cultivar): an *in vitro* and *in silico* investigations. Appl Biochem Biotechnol. (2022) 194:950–87. 10.1007/s12010-021-03669-8 34591254

[18] Sarkar T Salauddin M Pati S Sheikh HI Chakraborty R. Application of raw and differently dried pineapple (*Ananas comosus*) pulp on rasgulla (sweetened casein ball) to enhance its phenolic profile, shelf life, and in-vitro digestibility characteristics. J Food Process Preserv. (2021) 45:e15233. 10.1111/jfpp.15233

[19] Olewnik-Kruszkowska E Gierszewska M Richert A Grabska-Zielińska S Rudawska A Bouaziz M. Antibacterial films based on polylactide with the addition of quercetin and poly (ethylene glycol). Materials. (2021) 14:1643. 10.3390/ma14071643 33801625 PMC8036468

[20] Alugoju P Narsimulu D Bhanu JU Satyanarayana N Periyasamy L. Role of quercetin and caloric restriction on the biomolecular composition of aged rat cerebral cortex: an FTIR study. Spectrochim Acta A Mol Biomol Spectrosc. (2019) 220:117128. 10.1016/j.saa.2019.05.033 31146210

[21] Leite NB Martins DB Alvares DS dos Santos Cabrera MP. Quercetin induces lipid domain-dependent permeability. Chem Phys Lipids. (2022) 242:105160. 10.1016/j.chemphyslip.2021.105160 34808124

[22] Iwara IA Mboso EO Eteng OE Elot KN Igile GO Ebong PE. Peristrophebicalyculata extract and quercetin ameliorate high fat diet-streptozotocin-induced type ii diabetes in Wistar rats. Pharmacological Research-Modern Chinese Medicine. (2022) 2:100060. 10.1016/j.prmcm.2022.100060

[23] Jaishree V Narsimha S. Swertiamarin and quercetin combination ameliorates hyperglycemia, hyperlipidemia and oxidative stress in streptozotocin-induced type 2 diabetes mellitus in wistar rats. Biomed Pharmacother. (2020) 130:110561. 10.1016/j.biopha.2020.110561 32795923

[24] Mahabady MK Shamsi MM Ranjbar R Tabandeh MR Khazaeel K. Quercetin improved histological structure and upregulated adiponectin and adiponectin receptors in the placenta of rats with gestational diabetes mellitus. Placenta. (2021) 106:49–57. 10.1016/j.placenta.2021.02.008 33640737

[25] Nutmakul T. A review on benefits of quercetin in hyperuricemia and gouty arthritis. Saudi Pharm J. (2022) 30:918–26. 10.1016/j.jsps.2022.04.013 35903522 PMC9315272

[26] Tumova S Shi Y Carr IM Williamson G. Effects of quercetin and metabolites on uric acid biosynthesis and consequences for gene expression in the endothelium. Free Radic Biol Med. (2021) 162:191–201. 10.1016/j.freeradbiomed.2020.10.017 33091574

[27] Mazumder T Mamun IP Zaman MS Islam AK Chowdhury S Reza MS. Comparative lipid and uric acid suppressing properties of four common herbs in high fat-induced obese mice with their total phenolic and flavonoid index. Biochem Biophys Rep. (2021) 26:100990. 10.1016/j.bbrep.2021.100990 33869811 PMC8044636

[28] Durai L Kong CY Badhulika S. One-step solvothermal synthesis of nanoflake-nanorod WS2 hybrid for non-enzymatic detection of uric acid and quercetin in blood serum. Materials Science and Engineering: C. (2020) 107:110217. 10.1016/j.msec.2019.110217 31761166

[29] Aucoin M Cooley K Saunders PR Cardozo V Remy D Cramer H. The effect of quercetin on the prevention or treatment of COVID-19 and other respiratory tract infections in humans: a rapid review. Adv Integr Med. (2020) 7:247–51. 10.1016/j.aimed.2020.07.007 32837891 PMC7392107

[30] Shohan M Nashibi R Mahmoudian-Sani MR Abolnezhadian F Ghafourian M Alavi SM. The therapeutic efficacy of quercetin in combination with antiviral drugs in hospitalized COVID-19 patients: a randomized controlled trial. Eur J Pharmacol. (2022) 914:174615. 10.1016/j.ejphar.2021.174615 34863994 PMC8638148

[31] Manjunath SH Thimmulappa RK. Antiviral, immunomodulatory, and anticoagulant effects of quercetin and its derivatives: potential role in prevention and management of COVID-19. J Pharm Anal. (2021) 12:29–34. 10.1016/j.jpha.2021.09.009 34567823 PMC8450231

[32] Pawar A Pal A Goswami K Squitti R Rongiolettie M. Molecular basis of quercetin as a plausible common denominator of macrophage-cholesterol-fenofibrate dependent potential COVID-19 treatment axis. Results Chem. (2021) 3:100148. 10.1016/j.rechem.2021.100148 34150487 PMC8196513

[33] Mbikay M Chrétien M. Isoquercetin as an Anti-COVID-19 medication: a potential to realize. Front Pharmacol. (2022) 13:830205. 10.3389/fphar.2022.830205 35308240 PMC8924057

[34] Wang S Yao J Zhou B Yang J Chaudry MT Wang M. Bacteriostatic effect of quercetin as an antibiotic alternative *in vivo* and its antibacterial mechanism *in vitro*. J Food Prot. (2018) 81:68–78. 10.4315/0362-028X.JFP-17-214 29271686

[35] Hendra R Ahmad S Sukari A Shukor MY Oskoueian E. Flavonoid analyses and antimicrobial activity of various parts of *Phaleria macrocarpa* (Scheff) boerl fruit. Int J Mol Sci. (2011) 12:3422–31. 10.3390/ijms12063422 21747685 PMC3131569

[36] Liu HN Liu Y Hu LL Suo YL Zhang L Jin F. Effects of dietary supplementation of quercetin on performance, egg quality, cecal microflora populations, and antioxidant status in laying hens. Poult Sci. (2014) 93:347–53. 10.3382/ps.2013-03225 24570456

[37] Yang X Zhang W Zhao Z Li N Mou Z Sun D. Quercetin loading CdSe/ZnS nanoparticles as efficient antibacterial and anticancer materials. J Inorg Biochem. (2017) 167:36–48. 10.1016/j.jinorgbio.2016.11.023 27898345

[38] Zeng Y Nikitkova A Abdelsalam H Li J Xiao J. Activity of quercetin and kaemferol against *Streptococcus mutans* biofilm. Arch Oral Biol. (2019) 98:9–16. 10.1016/j.archoralbio.2018.11.005 30419487 PMC6555400

[39] Safwat NA Kashef MT Aziz RK Amer KF Ramadan MA. Quercetin 3-O-glucoside recovered from the wild Egyptian Sahara plant, Euphorbia paralias L, inhibits glutamine synthetase and has antimycobacterial activity. Tuberculosis. (2018) 108:106–13. 10.1016/j.tube.2017.11.005 29523309

[40] Das S Batra S Gupta PP Kumar M Srivastava VK Jyoti A. Identification and evaluation of quercetin as a potential inhibitor of naphthoate synthase from *Enterococcus faecalis*. J Mol Recognit. (2019) 32:e2802. 10.1002/jmr.2802 31353747

[41] Pan B Fang S Zhang J Pan Y Liu H Wang Y. Chinese herbal compounds against SARS-CoV-2: puerarin and quercetin impair the binding of viral S-protein to ACE2 receptor. Comput Struct Biotechnol J. (2020) 18:3518–27. 10.1016/j.csbj.2020.11.010 33200026 PMC7657012

[42] Ren M Wang X Hu M Jiang Y Xu D Xiang H. Enhanced bone formation in rat critical-size tibia defect by a novel quercetin-containing alpha-calcium sulphate hemihydrate/nano-hydroxyapatite composite. Biomed Pharmacother. (2022) 146:112570. 10.1016/j.biopha.2021.112570 34959114

[43] Kim HR Kim BM Won JY Lee KA Ko HM Kang YS. Quercetin, a plant polyphenol, has potential for the prevention of bone destruction in rheumatoid arthritis. J Med Food. (2019) 22:152–61. 10.1089/jmf.2018.4259 30596535

[44] Kim HS Ko MJ Park CH Chung MS. Application of pulsed electric field as a pre-treatment for subcritical water extraction of quercetin from onion skin. Foods. (2022) 11:1069. 10.3390/foods11081069 35454657 PMC9025617

[45] Nile A Gansukh E Park GS Kim DH Nile SH. Novel insights on the multi-functional properties of flavonol glucosides from red onion (*Allium cepa* L) solid waste–*In vitro* and *in silico* approach. Food Chem. (2021) 335:127650. 10.1016/j.foodchem.2020.127650 32745842

[46] Yang Y Zhang F. Ultrasound-assisted extraction of rutin and quercetin from *Euonymus alatus* (Thunb) sieb. Ultrasonics Sonochemistry. (2008) 15:308–13. 10.1016/j.ultsonch.2007.05.001 17606398

[47] Aghajanian S Kazemi S Esmaeili S Aghajanian S Moghadamnia AA. Sequential microwave-assisted extraction for isolation of quercetin from red kidney bean. Int J Eng. (2020) 33:12–7. 10.5829/ije.2020.33.01a.02

[48] Shi WM Xie J Wang Y. Determining the content of quercetin in *Medicage stativa* L. with enzyme extraction fluorescence method. Journal of Gannan Medical University. (2017) 2:1–15.

[49] Wong SK Chin KY Ima-Nirwana S. Quercetin as an agent for protecting the bone: a review of the current evidence. Int J Mol Sci. (2020) 21:6448. 10.3390/ijms21176448 32899435 PMC7503351

[50] Anachuna KK Moke GE Iyare C Katchy N Ben-Azu B Adeniyi B. Prenatal and early postnatal food restrictions cause changes in brain oxidative status and orexigenic/anorexigenic hormones in the offspring of rats: prevention by quercetin and kaempferol. Curr Res Pharmacol Drug Discov. (2020) 1:39–52. 10.1016/j.crphar.2020.100005 34909641 PMC8663934

[51] Grewal AK Singh TG Sharma D Sharma V Singh M Rahman MH. Mechanistic insights and perspectives involved in neuroprotective action of quercetin. Biomed Pharmacother. (2021) 140:111729. 10.1016/j.biopha.2021.111729 34044274

[52] Maiolini M Gause S Taylor J Steakin T Shipp G Lamichhane P. The war against tuberculosis: a review of natural compounds and their derivatives. Molecules. (2020) 25:3011. 10.3390/molecules25133011 32630150 PMC7412169

[53] Ferenczyova K Kalocayova B Bartekova M. Potential implications of quercetin and its derivatives in cardioprotection. Int J Mol Sci. (2020) 21:1585. 10.3390/ijms21051585 32111033 PMC7084176

[54] Langner E Lemieszek MK Rzeski W. Lycopene, sulforaphane, quercetin, and curcumin applied together show improved antiproliferative potential in colon cancer cells *in vitro*. J Food Biochem. (2019) 43:e12802. 10.1111/jfbc.12802 31353575

[55] Park DJ Jeon SJ Kang JB Koh PO. Quercetin reduces ischemic brain injury by preventing ischemia-induced decreases in the neuronal calcium sensor protein hippocalcin. Neuroscience. (2020) 430:47–62. 10.1016/j.neuroscience.2020.01.015 31982469

[56] Wu X Wang LJ Hou Y Guo RY Liu M Yang L. Different action mechanisms of low-and high-level quercetin in the brains of adult zebrafish (*Danio rerio*). Ecotoxicol Environ Saf. (2021) 223:112597. 10.1016/j.ecoenv.2021.112597 34365213

[57] Sharma D Rani R Chaturvedi M Rohilla P Yadav JP. In silico and in vitro approach of *Allium cepa* and isolated quercetin against MDR bacterial strains and *Mycobacterium smegmatis*. S Afr J Bot. (2019) 124:29–35. 10.1016/j.sajb.2019.04.019

[58] Khanfar E Nagy S Széchenyi A. Cocrystals of tuberculosis antibiotics: challenges and missed opportunities. Int J Pharm. (2022) 623:121924. 10.1016/j.ijpharm.2022.121924 35738333

[59] Shabbir M Afsar T Razak S Almajwal A Khan MR. Phytochemical analysis and evaluation of hepatoprotective effect of Maytenusroyleanus leaves extract against anti-tuberculosis drug induced liver injury in mice. Lipids Health Dis. (2020) 19:1–15. 10.1186/s12944-020-01231-9 32178678 PMC7077109

[60] Premalatha E Dineshraj R Kannan I Bhaarath KS Sharavanan TKV. Molecular docking study on quercetin derivatives as inhibitors of pantothenate synthetase (PanC) of *Mycobacterium tuberculosis*. Int J Res Pharm. (2020) 11:3684–90. 10.26452/ijrps.v11i3.2529

[61] Chaudhari VS Malakar TK Murty US Banerjee S. Extruded filaments derived 3D printed medicated skin patch to mitigate destructive pulmonary tuberculosis: design to delivery. Expert Opin Drug Deliv. (2021) 18:301–13. 10.1080/17425247.2021.1845648 33131339

[62] Pitaloka DAE Ramadhan DSF Chaidir L Fakih TM. Docking-based virtual screening and molecular dynamics simulations of quercetin analogs as enoyl-acyl carrier protein reductase (Inha) inhibitors of mycobacterium tuberculosis. Scientia Pharmaceutica. (2021) 89:20. 10.3390/scipharm89020020

[63] Guo G Gong L Sun L Xu H. Retracted article: quercetin supports cell viability and inhibits apoptosis in cardiocytes by down-regulating mir-199a. Artif Cells Nanomed Biotechnol. (2019) 47:2909–16. 10.1080/21691401.2019.1640711 31307244

[64] Bhat IUH Bhat R. Quercetin: a bioactive compound imparting cardiovascular and neuroprotective benefits: scope for exploring fresh produce, their wastes, and by-products. Biology. (2021) 10:586. 10.3390/biology10070586 34206761 PMC8301140

[65] Ulusoy HG Sanlier N. A minireview of quercetin: from its metabolism to possible mechanisms of its biological activities. Crit Rev Food Sci Nutr. (2020) 60:3290–303. 10.1080/10408398.2019.1683810 31680558

[66] Deng Q Li XX Fang Y Chen X Xue J. Therapeutic potential of quercetin as an antiatherosclerotic agent in atherosclerotic cardiovascular disease: a review. Evid-based Complement Altern Med. (2020) 2020:5926381. 10.1155/2020/5926381 32565865 PMC7292974

[67] Wang G Li Y Lei C Lei X Zhu X Yang L. Quercetin exerts antidepressant and cardioprotective effects in estrogen receptor α-deficient female mice *via* BDNF-AKT/ERK1/2 signaling. J Steroid Biochem Mol Biol. (2021) 206:105795. 10.1016/j.jsbmb.2020.105795 33246157

[68] Bostancieri N Elbe H Eşrefoglu M Vardi N. Cardioprotective potential of melatonin, quercetin and resveratrol in an experimental model of diabetes. Biotechnic and Histochemistry. (2022) 97:152–7. 10.1080/10520295.2021.1918766 33906539

[69] Reyes-Farias M Carrasco-Pozo C. The anti-cancer effect of quercetin: molecular implications in cancer metabolism. Int J Mol Sci. (2019) 20:3177. 10.3390/ijms20133177 31261749 PMC6651418

[70] Batiha GES Beshbishy AM Ikram M Mulla ZS El-Hack MEA Taha AE. The pharmacological activity, biochemical properties, and pharmacokinetics of the major natural polyphenolic flavonoid: quercetin. Foods. (2020) 9:374. 10.3390/foods9030374 32210182 PMC7143931

[71] Henidi HA Al-Abbasi FA El-Moselhy MA El-Bassossy HM Al-Abd AM. Despite blocking doxorubicin-induced vascular damage, quercetin ameliorates its antibreast cancer activity. Oxid Med Cell Longev. (2020) 2020:8157640. 10.1155/2020/8157640 33728016 PMC7939741

[72] Colpan RD Erdemir A. Co-delivery of quercetin and caffeic-acid phenethyl ester by polymeric nanoparticles for improved antitumor efficacy in colon cancer cells. J Microencapsul. (2021) 38:381–93. 10.1080/02652048.2021.1948623 34189998

[73] Sankaranarayanan R Sekhon PK Ambat A Nelson J Jose D Bhat GJ. Screening of human gut bacterial culture collection identifies species that biotransform quercetin into metabolites with anticancer properties. Int J Mol Sci. (2021) 22:7045. 10.3390/ijms22137045 34208885 PMC8269047

[74] Ezzati M Yousefi B Velaei K Safa A. A review on anti-cancer properties of quercetin in breast cancer. Life Sci. (2020) 248:117463. 10.1016/j.lfs.2020.117463 32097663

[75] Vafadar A Shabaninejad Z Movahedpour A Fallahi F Taghavipour M Ghasemi Y. Quercetin and cancer: new insights into its therapeutic effects on ovarian cancer cells. Cell Biosci. (2020) 10:1–17. 10.1186/s13578-020-00397-0 32175075 PMC7063794

[76] Shafabakhsh R Asemi Z. Quercetin: a natural compound for ovarian cancer treatment. J Ovarian Res. (2019) 12:1–9. 10.1186/s13048-019-0530-4 31202269 PMC6570913

